# Granite Powder vs. Fly Ash for the Sustainable Production of Air-Cured Cementitious Mortars

**DOI:** 10.3390/ma14051208

**Published:** 2021-03-04

**Authors:** Adrian Chajec

**Affiliations:** Department of Building Engineering, Wroclaw University of Science and Technology, 50-370 Wroclaw, Poland; adrian.chajec@pwr.edu.pl

**Keywords:** mineral powders, industrial wastes, economic analysis, sustainable development

## Abstract

The partial replacement of cement in concrete with the addition of granite powder and fly ash can help to reduce the carbon dioxide (CO_2_) emissions into the atmosphere associated with cement production. The aim of the article is to compare the performance of granite powder and fly ash for the sustainable production of air-cured cementitious mortars. The morphological, chemical, and granulometric properties of these additives were first compared with the properties of cement. Afterward, a series of mortars modified with the addition of granite powder and fly ash was made. The properties of the fresh mixes and the mechanical properties of the hardened composites were then tested. Finally, based on the obtained results, a cost analysis of the profitability of modifying cementitious composites with granite powder or fly ash was investigated. The obtained results allow similarities and differences between granite powder and fly ash in relation to cement to be shown. To conclude, it should be stated that both of these materials can successfully be used for the sustainable production of air-cured cementitious composites. This conclusion has a significant impact on the possibility of improving the natural environment by reducing the amount of cement production. More sustainable production of cement-based materials could enable CO_2_ emissions to be decreased. The use of granite powder for the production of cementitious mortars can significantly reduce the amount of this material deposited in landfills.

## 1. Introduction

Carbon dioxide (CO_2_) emissions into the atmosphere have been growing steadily for several decades [1,2]. Environmental protection has become one of the priority tasks for scientists around the world. Researchers are trying to find a method to reduce CO_2_ emissions into the atmosphere [3,4]. Cement can be defined as an adhesive substance that is capable of uniting fragments or masses of solid matter to a compact whole [5]. Cement production is one of the main causes of CO_2_ emissions in the world. This is due to the fact that cement is a key material used in the production of concrete [3,6]. When looking for opportunities to improve the natural environment, alternative material solutions should be tested (e.g., the use of marble and limestone powders) [7,8]. Scientists are trying to reduce the amount of cement in cementitious mixes by replacing it with supplementary cementitious materials (SCMs) [9,10,11,12,13,14,15]. Li et al. [9] described the filler technology that is related to the addition of supplementary cementitious materials such as mineral powders to cementitious composites. The authors observed that the addition of granite powder as a replacement for cement or cement paste could reduce the cement content in mixes. They stated that the method of replacing paste is a promising way of producing high-performance eco-friendly mortars, and it contributes to greater utilization of waste and lower cement content. A similar conclusion is described by Chen et al. [16]. Chu [17] described the effect of paste volume and w/c ratio on the properties of fresh and hardened concrete. He observed that modification of paste volume causes changes in fresh and hardened concrete properties. The author also suggested that decreasing paste volume (with an increase of mortar film thickness) could point to the direction of using filler technology for sustainable production of cementitious composites. Maddalena et al. [18] described the possibility of producing green cement with the sustainable use of SCMs such as metakaolin, silica fume, and nano-silica. Due to their high porosity and good thermal conductivity, carbon emissions in domestic construction could be reduced by 20–50%. D’Urso et al. [19] presented the new and innovative bio-inspired method of the advanced treatment of industrial sludge using a closed cycle drying process. They stated that this method could contribute to an increase in the number of hazardous wastes being used for the production of building materials. Puertas et al. [20] described the effect of using new raw mixes (e.g., fired red ceramic) and determined their reactivity and burnability when compared to clinker. They observed that it is possible to utilize these types of materials in cementitious mixes. All the authors mentioned in this paragraph stated that the use of SCMs could significantly improve sustainability in the construction industry. Supplementary cementitious materials can reduce the amount of cement in cementitious mixes without significantly changing their properties (mainly mechanical). One of the most commonly used SCMs in cementitious mixes is fly ash (FA) [21,22,23,24]. Giergiczny [23] described the most commonly used types of fly ashes—siliceous fly ash, calcium fly ash, and fluidized bed combustion fly ash. He also compared the methods of activation of fly ash and its impact on the properties of fresh and hardened cement mixes. Golewski [22] conducted research concerning the impact of fly ash and curing conditions on fracture toughness and the cracking mechanism in concrete. He observed that it is possible to make sustainable green concrete with the addition of fly ash with high mechanical properties. He also stated that fly ash decreases the early age strength of concrete and that the curing conditions have a significant impact on the properties of concrete with FA. This material is no longer waste and has become a product that is used in the production of cementitious mixtures [25]. This fact has led to the search for alternative materials that can reduce the amount of cement in cementitious mixes [26,27,28]. One of them is granite powder (GP) waste.

**Figure 1:**
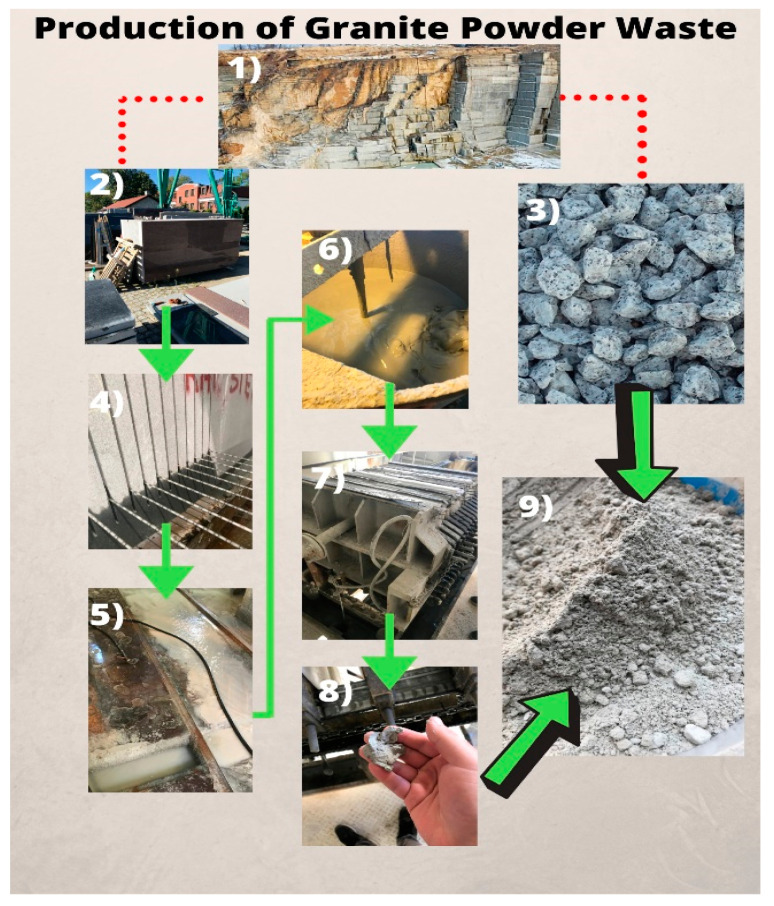
Production of granite powder waste.

Granite powder is a waste material generated when crushing or cutting granite rocks. Figure 1 shows the production process of granite powder waste. Due to the significant development of the stitching industry, granite powder waste deposits have increased in recent years, and this material currently has no significant application [29]. Granite powder waste has become a hazardous environmental problem. The possibilities of solving this issue are actively being sought [9,30,31,32].

Granite powder waste is a possible additive for cementitious mixes, but this requires more research. Attempts to use it in cementitious mixes have been going on for several years, and the number of studies conducted for cementitious mixes with this additive is constantly growing [33,34,35,36]. Gupta and Vyas [33] performed research related to the partial replacement of fine aggregate in cementitious mortars. They stated that the addition of granite powder (40%) improved the compressive strength of mortars, but at the same time, it decreased the bending tensile strength of the mortars. They also observed that the addition of granite powder increased the drying shrinkage and decreased the water absorption of the mortars. Sadek et al. [34] conducted research related to reusing granite powder in self-compacting concrete. They observed that the addition of granite powder causes an increase in the filler effect in composites. Moreover, the addition of granite powder in concrete could decrease the energy consumption that is used for operating vibrators and contribute to faster construction times. Granite powder, when compared to marble powder, exhibits a better influence on the properties of self-compacting concrete. Bakhoum et al. [35] conducted research related to the addition of granite powder nanoparticles in cementitious mortars. They observed that replacing 10% of cement with the nanoparticles of granite powders could increase the mechanical properties of composites. All of the above-mentioned authors concluded that granite powder has very good potential for use in the production of construction materials.

Fly ash is an additive that has been used for air-cured cementitious composites for many years, especially in air-cured cement-based materials (due to its significant increase in strength properties over a long period of time). However, in practice, problems with the availability of this material are increasingly encountered. The construction industry needs new materials that will allow a stable level of growth to be maintained [37,38,39,40]. Cementitious mortars are often used as building materials, and therefore the use of granite powder to produce them can be seen as a good idea [41,42]. Fly ash is the most widely used SCM, and its effect on air-cured cementitious mortars is known. Due to the fact that it is a well-recognized material, new materials that are meant to act in the same way should be compared with it. This approach is new concerning the study of the impact of granite powder on cementitious composites. Usually, the influence of the additive on the properties of the obtained composite is compared to the properties of the reference composite (unmodified). However, when using granite powder as SCM, its effect should be compared with that of the most commonly used SCM—fly ash.

Considering the above, it was decided that the purpose of this article is to compare the basic morphological and chemical characteristics of cement, fly ash, and granite powder. Additionally, it was decided to compare the influence of fly ash and granite powder on the mechanical properties of air-cured cementitious mortars. For a comprehensive analysis, a cost analysis related to the use of these materials in cement composites was also performed. The research conducted in this article may allow the impact of using waste mineral powders in cement composites to be compared with the effect of adding the most commonly used SCM, fly ash, and will therefore fulfill the research gap related to the use of waste mineral powders in cement composites. Currently, there is a lack of research results concerning granite powder waste and the analysis of its use in the construction industry.

## 2. Materials and Methods 

### 2.1. Designing of the Composite Mixes and the Preparation of Samples

The research concerned the properties of fresh and hardened air-cured composites. To achieve the goal of the research, seven different types of cementitious mortar mixes were prepared, the compositions of which differed from each other in terms of the used additive and its quantity. From these seven mixes, one mix was the reference mix that contained only ordinary Portland cement (denoted as Ref.), three mixes were made with the partial replacement of the ordinary Portland cement with siliceous fly ash in the proportions of 10%, 20%, and 30% (denoted as FA10, FA20, and FA30, respectively), and the other three mixes were made with the partial replacement of the ordinary Portland cement with waste granite powder–also in the proportions of 10%, 20%, and 30% (denoted as GP10, GP20, and GP30, respectively). The series of mortar mixes are listed in Table 1.

CEM I 42.5 cement was used in the research, the composition of which included 95–100% of Portland clinker, and 0–5% of secondary components. Its compressive strength was as follows: early ≥20 MPa, and standard ≥42.5 MPa and ≤62.5 MPa. Its other properties included setting time (beginning) ≥60 min, loss of ignition ≤5%, volume stability ≤10 mm, the content of SO_3_ ≤4%, and the content of chloride ≤0.1% (information from the manufacturer). The used siliceous fly ash had a specific density of about 2100 kg/m^3^, and reactivity after 28 days ≥75%, and after 90 days ≥85%. Other properties included the content of CaO ≤10%, the content of SiO_2_ ≥25%, and volume stability ≤10 mm (information from the manufacturer). The sand that was used in the tests had a specific density of about 1600 kg/m^3^, and absorptivity W_A,24_ = 0.6%. Granular analysis of the binders and fine aggregate was performed according to procedure PN-EN 993-1 [43]. The samples were prepared according to the procedure [44] and were then cured for the required time (7 days, 28 days, or 90 days). Afterward, the samples were tested with regard to their strength properties using destructive tests. The samples were air-cured at a temperature of 20–23 °C and a humidity of 60–65%.

### 2.2. Determination of the Basic Properties of Binders

The basic morphological properties of the binders were determined using scanning electron microscopy. The sieve size distributions and the specific surface area were obtained using the Blaine apparatus (ELE International, Kowloon, Hong Kong, China). The basic chemical properties were determined using X-ray diffraction analysis (Bruker, Ettlingen, Germany).

#### 2.2.1. Morphological Properties

The properties of the binders depend on the shape of their grains [45,46]. The basic properties that are required for the morphological analysis of the tested material’s grains can be determined using images from a scanning electron microscope. Pictures were taken using an accelerating voltage of 20 kV at approximately 500×. The basic morphological properties of the grains include the length (*L*) and width (*W*) of its 2D projection. These parameters describe a relationship called the aspect ratio (*AR*).

(1) $$AR=\frac{L}{W}[-]$$

The value of the aspect ratio is a basic parameter, which describes the morphological properties of aggregate. If the aspect ratio value is close to 1.0, it means that the shape of the aggregate’s grain is close to that of a circle. The more the *AR* value differs from 1.0, the more elongated the shape is.

Roundness (*R*) is a parameter describing the relationship between the grain’s length (*l*) and the surface of this grain (*A*_1_), which was designated from its 2D projection.

(2) $$R=\frac{l^{2}}{4\pi A_{1}}[-]$$

The area ratio (*Ar*) describes the relationship between the aggregate grain surface (2D projection) and the ellipse surface in which this projection can be entered.

(3) $$A_{r}=\frac{A_{ce}}{A_{1}}[-]$$

**Figure 2:**
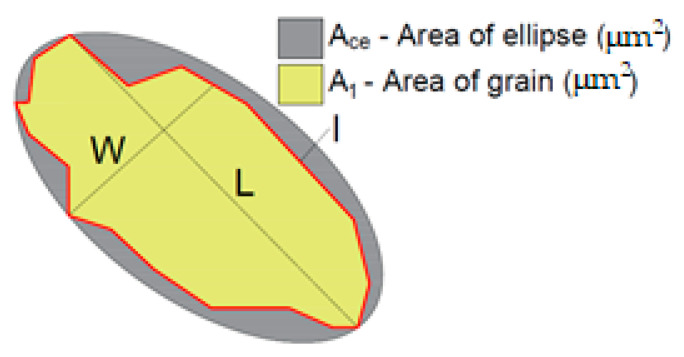
Basic parameters used for the morphological grain analysis.

Figure 2 presents the individual parameters necessary to perform the analysis.

#### 2.2.2. Chemical Properties

**Figure 3:**
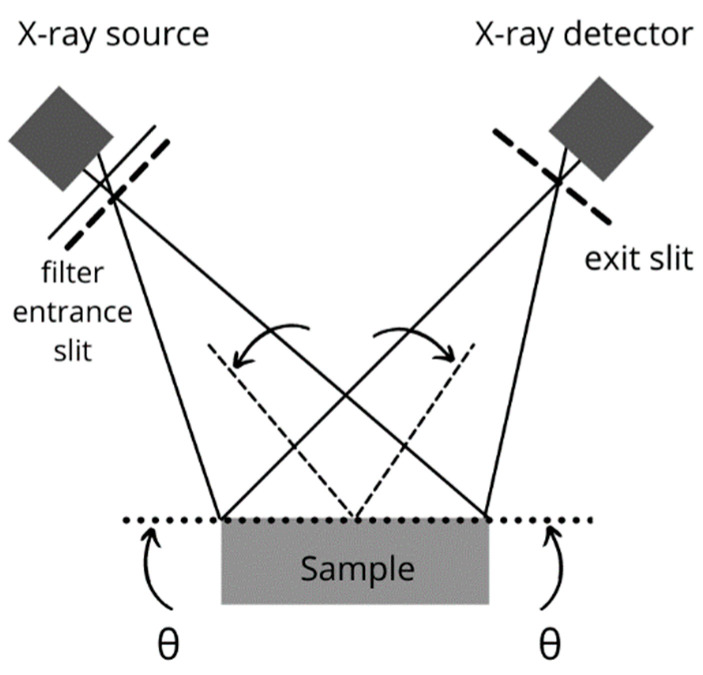
Scheme of testing using the X-ray diffraction method.

The chemical properties of the binders were determined based on X-ray diffraction analysis. This test is presented in Figure 3. During the test, the device determines the atoms in the chemical composition and then analyzes the number of individual atoms in it. The test allows the content of the chemical compounds (oxides) in the analyzed substance to be determined.

### 2.3. Determination of the Consistency of the Fresh Mortars

The consistency of the fresh cementitious mortar mixes was determined based on the Novikov cone subsidence. The method involves placing the mortar in a measuring bucket in two stages after which each layer is compacted [47]. After placing the mix in the measuring bucket, the Novikov cone was placed in its center so that its end was touching the surface of the mortar. It was then released in such a way that it could fall vertically downward. After 10 s, the cone was removed and the depth of its immersion in the mortar was determined. Based on its immersion, the consistency of the mortar was designated and then compared with the mortars with different compositions. The tests were carried out in accordance with the requirements set out in procedure PN-B-04500:1985 [48].

### 2.4. Determination of the Basic Physical Properties of the Hardened Mortars

The basic physical properties of the hardened mortar, such as bulk density and water absorption, were determined. The tests were carried out in accordance with the requirements set out in procedure PN-B-04500:1985 [48].

The determination of bulk density involves drying the samples to a constant volumetric weight (difference in measurements after 24 h <1.0% of the weight), followed by accurately measuring the sample’s dimensions. The bulk density of the sample is then determined.

**Figure 4:**
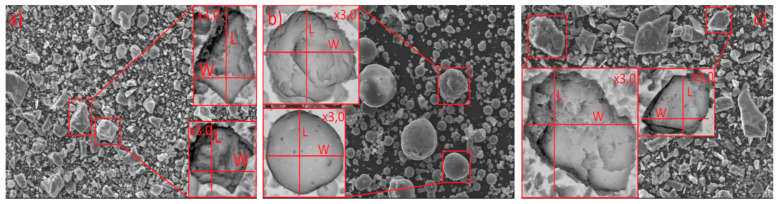
Scanning electron microscope (SEM) analysis of the binders: (**a**) cement CEM I 42.5, (**b**) siliceous fly ash, and (**c**) granite powder.

To determine water absorption (*W_a_*), the samples for which bulk density was determined were placed in water and then weighed every 24 h to determine when the samples were fully saturated (difference in sample weight <1.0%). After determining the amount of water absorbed by the samples, the water absorption was calculated. Water absorption is determined on the basis of the dry sample’s mass (*m_d_*) and the wet sample’s mass (*m_w_*). Figure 4 shows photos of the testing of the water absorption of the samples.

(4) $$W_{a}=\frac{m_{w}-m_{d}}{m_{d}}\times 100\%\;[\%]$$

Volume porosity (*P_v_*) is determined on the basis of the ratio of the air pore volume (*V_p_*) in the tested material to the volume (*V*) of the tested sample.

(5) $$P_{v}=\frac{V_{p}}{V}\times 100\%\;[\%]\;$$

### 2.5. Determination of the Basic Mechanical Properties of the Hardened Mortars

To determine the basic mechanical properties of the hardened mortars, destructive testing of the samples was performed in terms of the compressive strength test and the bending tensile strength test. Air-cured samples—beams with the dimensions of 40 mm × 40 mm × 160 mm—were first subjected to the bending tensile strength test after 72,890 days. Afterward, their two halves (with the dimensions of 40 mm × 40 mm × 80 mm) were subjected to the compressive strength test. The tests were performed using a testing machine in a laboratory at Wroclaw University of Science and Technology (Wrocław, Poland). The tests were carried out in accordance with the requirements set out in procedure PN-EN 196-1 [44] and PN-EN 1015-11 [49]. Figure 4 presents photos of testing the basic mechanical properties of the hardened mortars.

Brittleness (*B*) is a parameter describing the ratio of the basic mechanical properties of hardened cementitious mortars. If the brittleness value is less than 0.125 [–], then the mortar is not very brittle, and if the brittleness value is greater than 0.125 [–], then the mortar is very brittle.

(6) $$B=\frac{f_{ctm}}{f_{cm}}\;[-]$$

## 3. Results

### 3.1. Determination of the Basic Properties of the Binders

#### 3.1.1. Determination of the Basic Morphological Properties of the Binders

**Figure 5:**
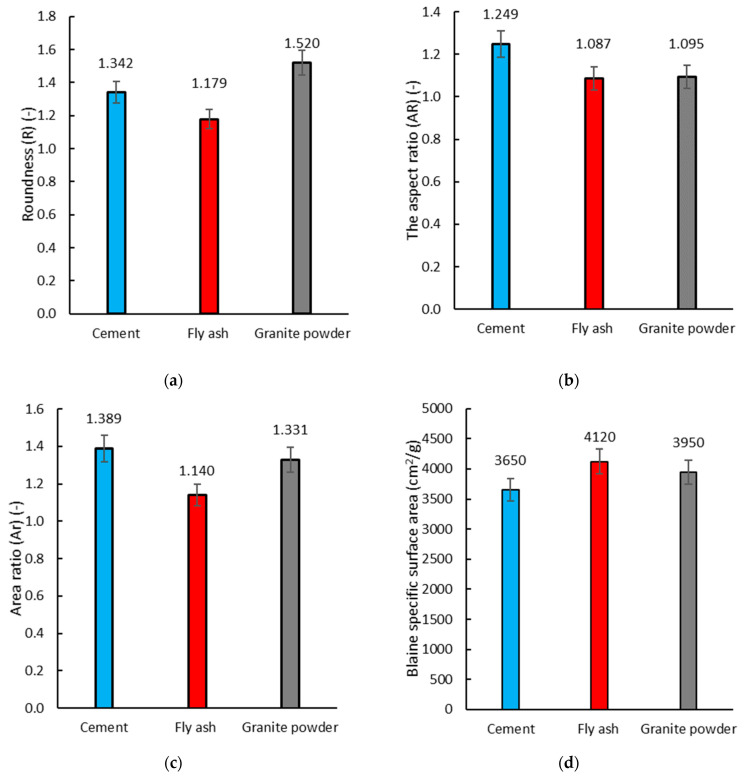
Morphological analysis of the grains of the binders: (**a**) the area ratio (*Ar*), (**b**) the aspect ratio (*AR*), (**c**) roundness (*R*), and (**d**) the specific surface area (Blaine).

The basic morphological properties of the binders were determined using scanning electron microscopy. The sieve size distributions and the specific surface area were obtained using the Blaine apparatus. Figure 4 shows images from the scanning electron microscope (SEM). The images made it possible to recognize the characteristics of the graining and grain morphology in the cement (a), siliceous fly ash (b), and granite powder (c). Figure 5 shows the obtained results of the analysis of the morphological properties.

**Figure 6:**
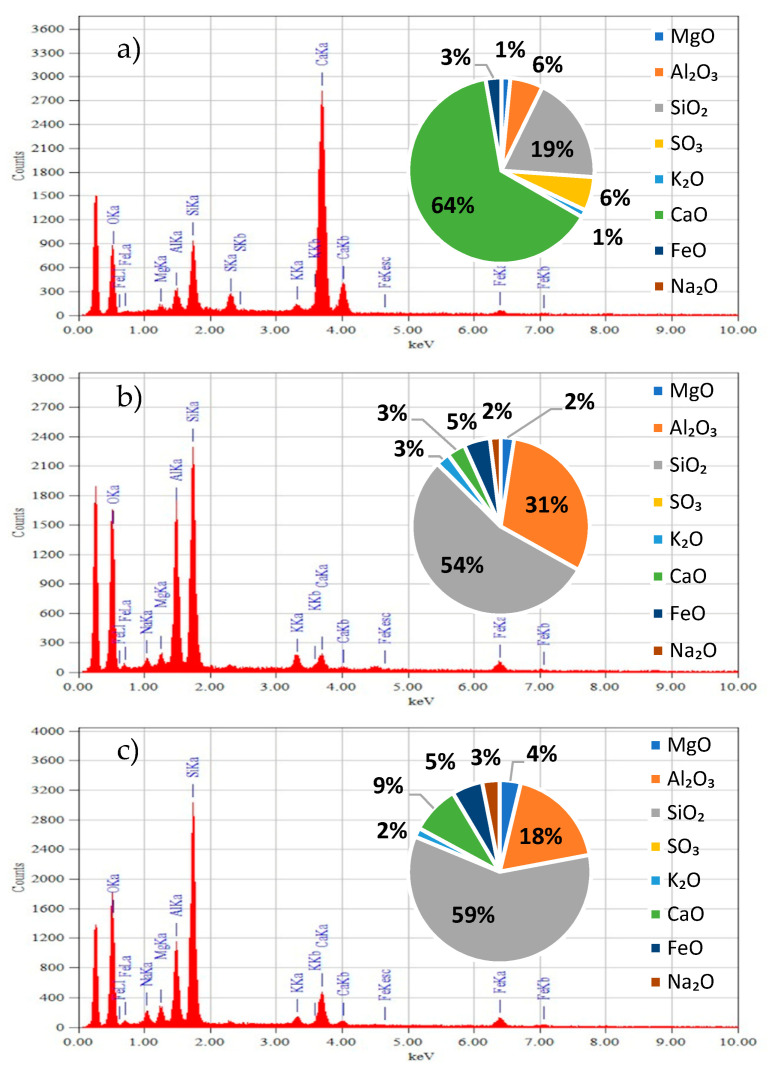
X-ray diffraction chemical composition analysis: (**a**) cement, (**b**) fly ash, and (**c**) granite powder.

Figure 5 shows that there are differences in the microstructure of the individual binders. The granite powder has a similar structure to the cement, while the fly ash has a completely different structure than the cement and granite powder. Overall, 15 measurements were conducted for each component. The results shown in Figure 6 represent the mean values. Figure 5a shows the roundness (*R*) value of the individual binders. The granite powder has the highest roundness (*R*) value, while the fly ash has the lowest value of this parameter. In the case of the roundness of the granite powder and fly ash, there is a difference of about 27%, while the roundness value of the cement is smaller than that of the granite powder by about 12%. Figure 5b shows the aspect ratio (*AR*) parameter. It can be seen that its value for the granite powder and fly ash is similar, while the aspect ratio (*AR*) value for the cement is about 20% more than for the other two binders. Figure 5c shows the area ratio (*Ar*) parameter. The value of the area ratio (*Ar*) for the granite powder is similar to the cement, while the value of the area ratio (*Ar*) for the fly ash is about 20% lower than that of the other two binders. Figure 5d shows the value of the specific surface area (Blaine). The value of the specific surface area for the granite powder is greater than the specific surface area of the cement by about 10%. The specific surface area of the fly ash is the largest and is greater than the specific surface area of the granite powder by about 5%. The results obtained from the analysis of the morphology of the binder grains indicate, as was the case in other studies [50,51], that the properties of cementitious mixes with additives will vary.

#### 3.1.2. Chemical Properties of the Binders

The basic chemical properties of the binders were determined using X-ray diffraction analysis. The X-ray diffraction test involved the determination of the chemical composition of the compounds. Figure 6 shows the results of the study for the cement (Figure 6a), fly ash (Figure 6b), and granite powder (Figure 6c).

Figure 6 shows the results of the chemical composition analysis of the binders. The chemical composition of the granite powder (Figure 6c) and fly ash (Figure 6b) is similar (chemical compounds present), but the content of the individual chemical compounds differs. The granite powder and fly ash have a different chemical composition when compared to the cement (Figure 6a). Fly ash and granite powder have a similar amount of SiO_2_ (54% and 59%, respectively). In turn, cement has a lower SiO_2_ content (6%). Fly ash and granite powder have a low CaO content of <10%, in contrast to cement (64%). It was observed that each of the analyzed materials has a different Al_2_O_3_ content (6%, 31%, and 18%, respectively).

#### 3.1.3. Granular Analysis of the Binders and Fine Aggregate

**Figure 7:**
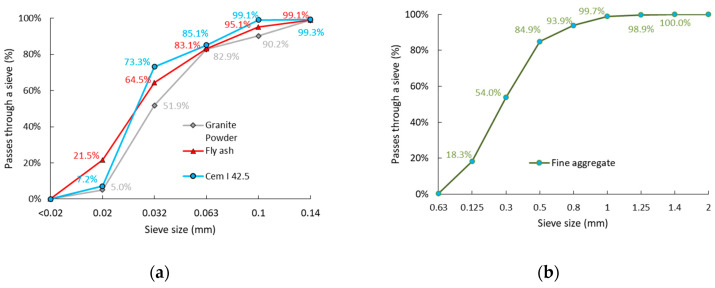
Sieve size development for the (**a**) binders and (**b**) fine aggregate that were used in the research.

When analyzing Figure 7a, it should be stated that the grain size of all the binders is similar. However, the distribution of grains with a size smaller than 0.063 mm differs. The cement has a large amount (about 65%) of grains of 0.032–0.02 mm, while the granite powder has about 45%, and the fly ash has about 43%. It should also be mentioned that the fly ash has a high content of grains <0.02 mm (about 21%), while the cement has about 7%, and the granite powder about 5%. Figure 7b presents the sieve size development for the sand used in the research.

### 3.2. The Consistency of the Mortars

**Figure 8:**
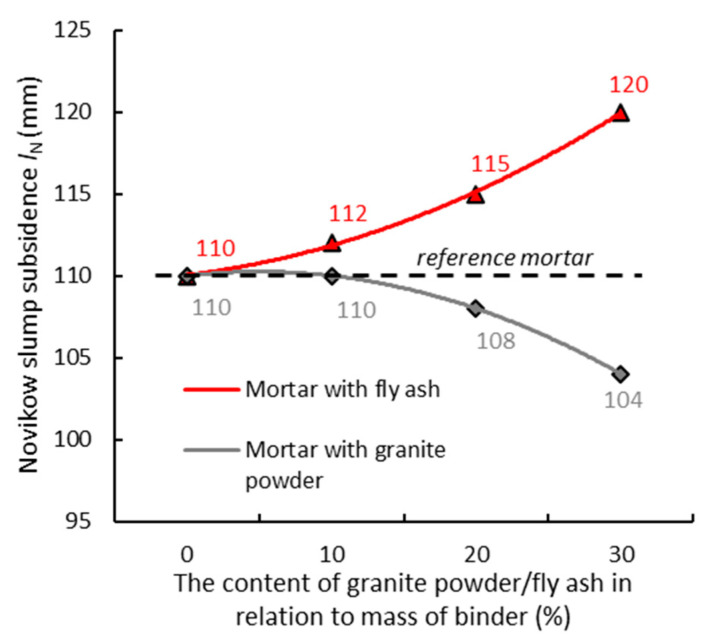
Test results of the Novikov cone subsidence (*l_N_*) for the mortars, which differ regarding their content of granite powder/fly ash in relation to the mass of the cement.

Figure 8 shows the test results of the Novikov cone subsidence (*l_N_*) for the mortars, which differ regarding their content of granite powder/fly ash in relation to the mass of the cement.

Figure 8 shows that with the increase in the content of granite powder in the mortar, the Novikov slump subsidence (*l_N_*) of the mortar is lower in relation to the reference mortar (maximum by about 6%). It is evident from Figure 8 that with the increase in the content of fly ash in the mortar, the Novikov slump subsidence (*l_N_*) of the mortar slightly increases (maximum by about 10%). This may be due to the fact that the shape and morphology of the fly ash grains cause a change in the consistency of the cementitious mix, which becomes more fluid. A similar relationship was observed in other studies in the cases of granite powder [52,53] and fly ash [54,55].

### 3.3. Results of the Basic Physical Properties of the Air-Cured Hardened Composites 

**Figure 9:**
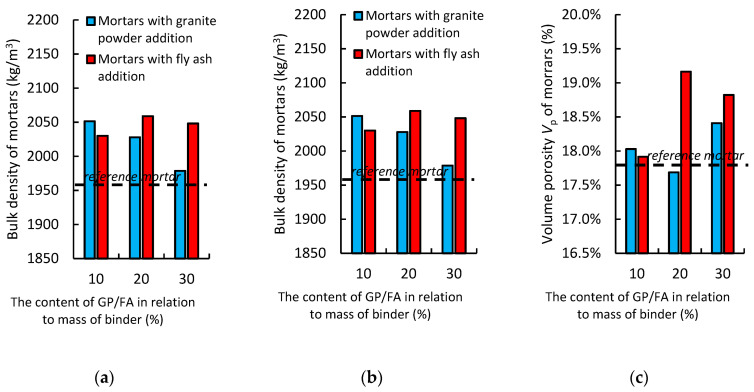
The effect of replacing the cement with the granite powder or fly ash on the following basic physical properties: (**a**) bulk density, (**b**) water absorption, and (**c**) volume porosity of the air-cured hardened cementitious mortars.

The determination of the basic physical properties of the hardened composites involved testing the bulk density of the composites and their absorptivity. Figure 9 presents the results of these tests. The type and amount of additive, when compared to the reference value, are provided in the graphs.

Figure 9a shows the effect of replacing the cement with granite powder or fly ash on the bulk density of the air-cured hardened cementitious composites. The mortar with the addition of the granite powder has a higher bulk density than the reference mortar (about 4%), and its volume density decreases with an increase in the amount of cement being replaced with granite powder. The mortar with the addition of the fly ash also has a higher volume density than the reference mortar (about 4%), while its volume density increases with an increase in the replacement of the cement with the fly ash.

Figure 9b shows the effect of replacing the cement with granite powder or fly ash on the water absorption (*W_a_*) of the air-cured hardened cementitious composites. The mortar with the addition of the granite powder has a higher water absorption (*W_a_*) value than the reference mortar (by about 0.5%), and the value of water absorption (*W_a_*) increases as the amount of cement replaced by the granite powder increases. The mortar with the addition of the fly ash has a lower water absorption (*W_a_*) value than the reference mortar (by about 0.3%), and the value of water absorption (*W_a_*) decreases as the amount of cement replaced by the fly ash decreases.

Figure 9c shows the effect of replacing a portion of cement with granite powder or fly ash on the volume porosity (*P_v_*) of the air-cured hardened cementitious composites. The mortar with the addition of the granite powder has a higher volume porosity (*P_v_*) than the reference mortar (by about 0.5%), and the value of volume porosity (*P_v_*) increases as the amount of cement being replaced by the granite powder increases. The mortar with the addition of the fly ash has a higher volume porosity (*P_v_*) than the reference mortar (by about 1.0%), and the value of volume porosity (*P_v_*) increases as the amount of cement being replaced by the fly ash increases.

The differences in the physical properties may be related to the fact that the granite powder waste and fly ash differ significantly in terms of their grain structure and the parameters describing their surface morphology. In addition, there is a significant difference regarding their surface areas. It is also important that granite powder does not have pozzolanic properties, and fly ash does. A similar relationship was observed in research conducted by Singh [52] and Gupta [33].

### 3.4. Results of the Basic Mechanical Properties of the Air-Cured Hardened Composites

**Figure 10:**
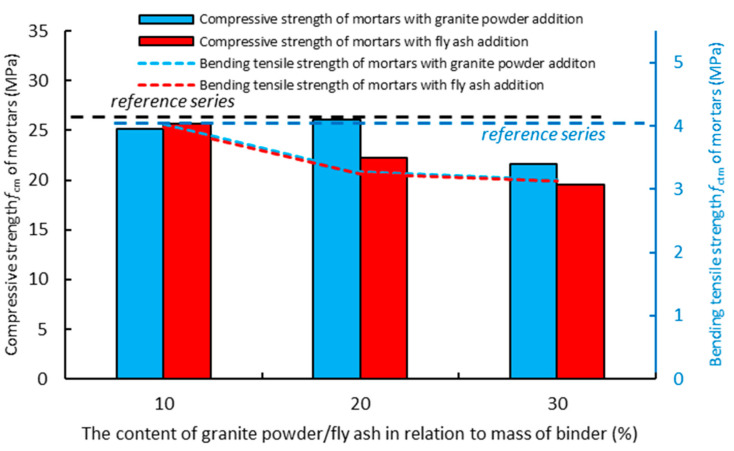
The effect of replacing the cement with the granite powder or fly ash on the compressive strength (*f_cm_*) and bending tensile strength (*f_ctm_*) of the air-cured hardened cementitious composites after seven days.

The determination of the basic mechanical properties of the air-cured hardened composites involved the conducting of destructive tests—the compressive strength and bending tensile strength tests—in a timely manner (7 days, 28 days, or 90 days from the moment of forming the samples). Figure 10 presents the results of determining the compressive strength and bending tensile strength for the air-cured composites modified with the addition of the granite powder and fly ash. The results were compared with the values of the reference mortar (without additives).

Figure 10 shows the effect of replacing the cement with the granite powder or fly ash on the compressive strength (*f_cm_*) of the air-cured hardened cementitious composites after seven days. The mortar with the addition of the granite powder has a lower value of compressive strength (*f_cm_*) than the reference mortar, and its value decreases as the amount of cement replaced by the granite powder increases. The mortar with the addition of the fly ash also has a lower value of compressive strength (*f_cm_*) than the reference mortar, and its value also decreases as the amount of cement being replaced by the fly ash increases. The composites with the addition of the granite powder and fly ash have similar values of bending tensile strength (*f_ctm_*). These values are lower than the value of bending tensile strength (*f_ctm_*) for the reference mortar. The bending tensile strength decreases with an increase in the amount of cement being replaced by the additive.

**Figure 11:**
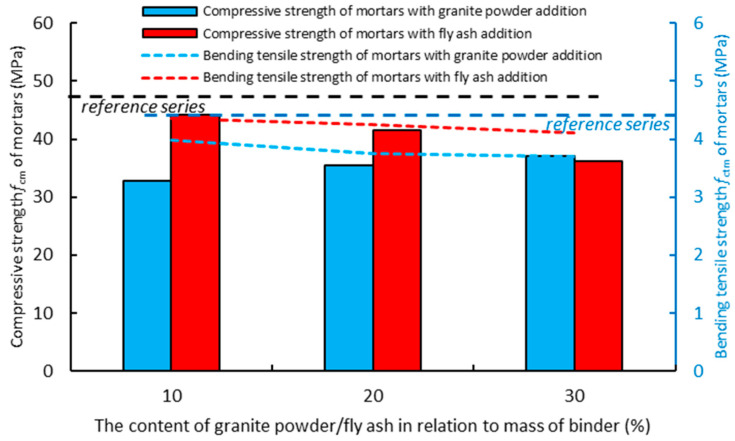
The effect of replacing the cement with the granite powder or fly ash on the compressive strength (*f_cm_*) and bending tensile strength (*f_ctm_*) of the air-cured hardened cementitious composites after 28 days.

Figure 11 shows the effect of replacing the cement with the granite powder or fly ash on the compressive strength (*f_cm_*) of the air-cured hardened cementitious composites after 28 days. The mortar with the addition of the granite powder has a lower value of compressive strength (*f_cm_*) than the reference mortar, and its value does not change significantly with the increase in the replacement of the cement with the granite powder. The mortar with the addition of the fly ash also has a lower compressive strength (*f_cm_*) value than the reference mortar, and its value also decreases as the amount of cement being replaced by the fly ash increases. The mortar with the addition of the granite powder has on average about a 12% lower value of compressive strength (*f_cm_*) than the mortar with the addition of the fly ash. The composite with the addition of the granite powder has a lower value than the reference mortar, and its value decreases as the amount of cement being replaced by the granite powder increases. The mortar with the addition of the fly ash also has a lower value of bending tensile strength (*f_ctm_*) than the reference mortar, and its value decreases as the amount of cement being replaced by the fly ash increases. The mortar with the granite powder has a lower bending tensile strength (*f_ctm_*) value than the mortar with the fly ash by about 10%.

**Figure 12:**
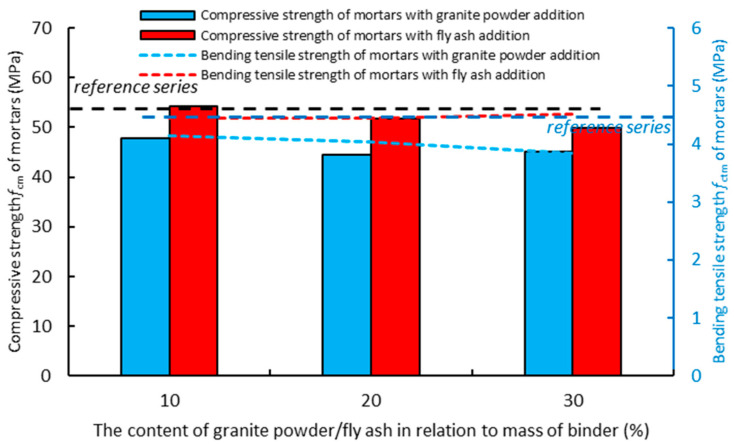
The effect of replacing the cement with the granite powder or fly ash on the compressive strength (fcm) and bending tensile strength (fctm) of the air-cured hardened cementitious composites after 90 days.

Figure 12 shows the effect of replacing the cement with the granite powder or fly ash on the compressive strength (*f_cm_*) of the air-cured hardened cementitious composites after 90 days. The mortar with the addition of the granite powder has a lower value of compressive strength (*f_cm_*) than the reference mortar, and its value does not change significantly with the increase in the replacement of the cement by the granite powder. The mortar with the addition of the fly ash has a similar value of compressive strength (*f_cm_*) to the reference mortar, and its value decreases as the amount of cement being replaced by the fly ash increases. The mortar with the addition of the granite powder has on average about a 15% lower compressive strength (*f_cm_*) than the mortar with the addition of the fly ash. The mortar with the addition of the granite powder has a lower value than the reference mortar, and its value decreases as the amount of cement being replaced by the granite powder increases. The mortar with the addition of the fly ash has a similar value of bending tensile strength (*f_ctm_*) to the reference mortar, and its value increases slightly as the amount of cement being replaced by the fly ash increases. The mortar with the granite powder has a lower bending tensile strength (*f_ctm_*) value than the mortar with the fly ash by about 10%.

**Figure 13:**
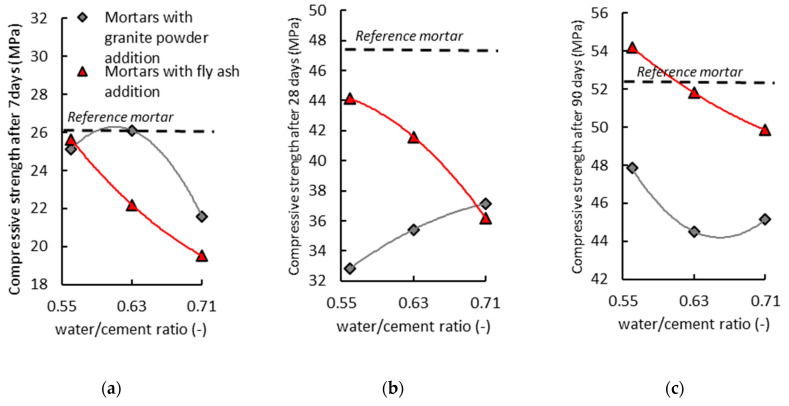
The effect of the water/cement ratio (w/c) on the compressive strength (*f_cm_*) of the air-cured hardened cementitious mortars: (**a**) after 7 days, (**b**) after 28 days, and (**c**) after 90 days.

Figure 13a shows the effect of the water/cement ratio (w/c) on the compressive strength (*f_cm_*) of the air-cured hardened cementitious mortars after seven days. The mortar with the addition of the granite powder has a similar value of compressive strength (*f_cm_*) to the reference mortar, and its value decreases as the w/c parameter increases. The mortar with the addition of the fly ash has a lower compressive strength (*f_cm_*) value than the reference mortar, and its value decreases as the w/c parameter increases. The mortar with the granite powder on average has about a 20% higher compressive strength (*f_cm_*) value than the mortar with the fly ash. 

Figure 13b shows the effect of the water/cement ratio (w/c) on the compressive strength (*f_cm_*) of the air-cured hardened cementitious mortars after 28 days. The mortar with the addition of the granite powder has a lower value of compressive strength (*f_cm_*) than the reference mortar, and its value does not change significantly with the increase of the w/c parameter. The mortar with the addition of the fly ash has a lower compressive strength (*f_cm_*) value than the reference mortar, and its value decreases as the w/c parameter increases. The mortar with the addition of the granite powder on average has about an 18% lower value of compressive strength (*f_cm_*) than the mortar with the addition of the fly ash.

Figure 13c shows the effect of the water/cement ratio (w/c) on the compressive strength (*f_cm_*) of the air-cured hardened cementitious mortars after 90 days. The mortar with the addition of the granite powder has a lower value of compressive strength (*f_cm_*) than the reference mortar, and its value does not change significantly with the increase of the w/c parameter. The mortar with the addition of the fly ash has a similar value of compressive strength (*f_cm_*) to the reference mortar, and its value slightly decreases as the w/c parameter increases. The mortar with the granite powder on average has about a 14% lower compressive strength (*f_cm_*) value than the mortar with the fly ash.

**Figure 14:**
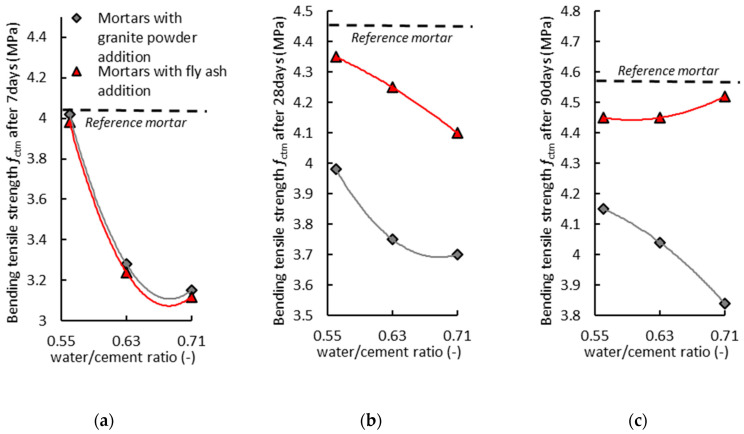
The effect of the water/cement ratio (w/c) value on the bending tensile strength (*f_ctm_*) of the air-cured hardened cementitious mortars: (**a**) after 7 days, (**b**) after 28 days, and (**c**) after 90 days.

Figure 14a shows the effect of the water/cement ratio (w/c) value on the bending tensile strength (*f_ctm_*) of the air-cured hardened cementitious mortars after seven days. The mortar with the addition of the granite powder has a lower value of bending tensile strength (*f_ctm_*) than the reference mortar, and its value decreases as the w/c parameter increases. The mortar with the addition of the fly ash has a lower bending tensile strength (*f_ctm_*) value than the reference mortar, and its value decreases as the w/c parameter increases. The mortar with the addition of the granite powder and fly ash has similar values.

Figure 14b shows the effect of the water/cement ratio (w/c) value on the bending tensile strength (*f_ctm_*) of the air-cured hardened cementitious mortars after 28 days. The mortar with the addition of the granite powder has a lower value of bending tensile strength (*f_ctm_*) than the reference mortar, and its value decreases as the w/c parameter increases. The mortar with the addition of the fly ash has a lower bending tensile strength (*f_ctm_*) value than the reference mortar, and its value decreases as the w/c parameter increases. The mortar with the granite powder has on average about a 20% lower bending tensile strength (*f_ctm_*) value than the mortar with the fly ash.

Figure 14c shows the effect of the water/cement ratio (w/c) value on the bending tensile strength (*f_ctm_*) of the air-cured hardened cementitious mortars after 90 days. The mortar with the addition of the granite powder has a lower value of bending tensile strength (*f_ctm_*) than the reference mortar, and its value does not change significantly with the increase of the w/c parameter. The mortar with the addition of the fly ash has a similar bending tensile strength (*f_ctm_*) value to the reference mortar, and its value does not change significantly with the increase of the w/c parameter. The mortar with the granite powder has on average about a 10% lower bending tensile strength (*f_ctm_*) value than the mortar with the fly ash.

**Figure 15:**
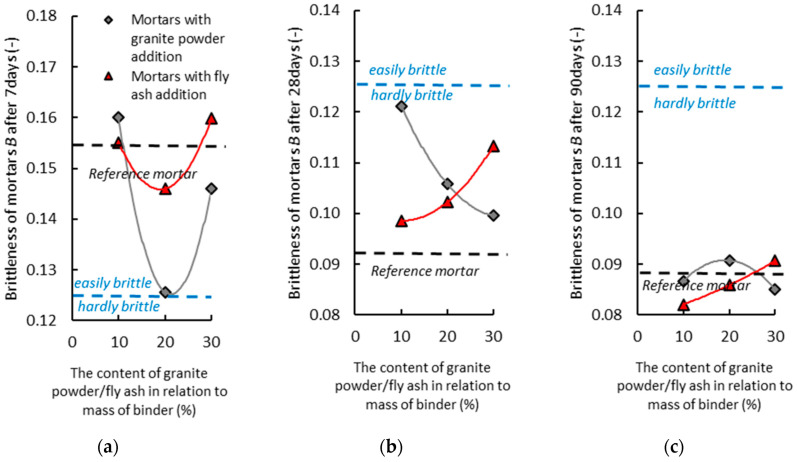
The effect of the amount of cement being replaced with the granite powder or fly ash on the brittleness of the air-cured hardened cementitious mortars: (**a**) after 7 days, (**b**) after 28 days, (**c**) after 90 days.

Figure 15a shows the effect of the amount of cement being replaced with the granite powder or fly ash on the brittleness of the air-cured hardened cementitious mortars after seven days. The mortar with the addition of the granite powder has a lower value of brittleness than the reference mortar, and its value decreases slightly as the amount of granite powder increases. The mortar with the addition of the granite powder is also very brittle. The mortar with the addition of the fly ash has a similar value of brittleness to the reference mortar, and its value does not change significantly with the increase in the amount of the additive. The mortar with the addition of the fly ash is very brittle. The mortar with the addition of the granite powder has a lower value of brittleness than the mortar with the fly ash.

Figure 15b shows the effect of the amount of cement being replaced with the granite powder or fly ash on the brittleness of the air-cured hardened cementitious mortars after 28 days. The mortar with the addition of the granite powder has a higher value of brittleness than the reference mortar, and its value decreases slightly as the amount of granite powder increases. The mortar with the addition of the granite powder is not very brittle. The mortar with the addition of the fly ash has a higher value of brittleness than the reference mortar, and its value does not change significantly with the increase in the amount of the additive. The mortar with the addition of the fly ash is not very brittle. The mortar with the addition of the granite powder has a similar value of brittleness as the mortar with the fly ash.

Figure 15c shows the effect of the amount of cement being replaced with the granite powder or fly ash on the brittleness of the air-cured hardened cementitious mortars after 90 days. The mortar with the addition of the granite powder has a higher value of brittleness than the reference mortar, and its value does not change significantly with the increase in the amount of granite powder. The mortar with the addition of the granite powder is not very brittle. The mortar with the addition of the fly ash has a similar value of brittleness to the reference mortar, and its value does not change significantly with the increase in the amount of the additive. The mortar with the addition of the fly ash is not very brittle. The mortar with the addition of the granite powder has a similar value of brittleness to the mortar with the fly ash.

The differences between the results of the basic mechanical properties of the air-cured hardened mortars may be related to the fact that the granite powder has a smaller specific surface area than the fly ash and a smaller number of silty grains. This has a direct impact on the water demand of the cementitious mix, with water being necessary for the proper cement hydration process. In addition, the study in [23] reported that the pozzolanic properties of fly ash reduce the early (seven days) compressive strength and bending tensile strength of mortars. However, compressive strength and bending tensile strength increase after 90 days of curing. A similar phenomenon was observed in the case of granite powder in research [9,56,57].

## 4. Economic Analysis of the Potential Use of Granite Powder vs. Fly Ash for the Production of Air-Cured Cementitious Mortars

Today, increasing attention is paid to the cost of making a cementitious mix. Both cement and aggregate prices are currently at such a high level that researchers from around the world are trying to find alternative materials or materials that will reduce the amount of cement or aggregate in mixes. However, cost optimization should always refer to the mechanical properties of the mixes. A cementitious mix designer should not strive to achieve the best price-performance ratio when choosing a mix. To facilitate this assessment, based on the performed tests, a mechanical and economic analysis related to the addition of fly ash and granite powder is presented below. The analysis was carried out in two stages. The first involved the analysis of the mechanical properties with regards to the type of additive and its quantity, followed by the calculation of the mechanical performance ratio (*MPR*) [58]. The second stage of the analysis was the determination of the cost of the cementitious mix with regards to the additive and its amount, and the carrying out of calculations of the effective cost ratio (*ECR*). The summary of the analysis shows a comparison of the *MPR* and *ECR* coefficients with regards to the type of additive and its quantity.

### 4.1. Analysis of the Mechanical Performance of the Cementitious Mortars

Section 3.4 describes the results of the compressive strength and bending tensile strength of the cementitious mortars with regards to the additives and their amounts. To compare the obtained results, a mechanical performance ratio (*MPR*) [58] was used as follows:

(7) $$MPR=\frac{4\times \frac{f_{cm,mixes}}{f_{cm,ref}}+2\times \frac{f_{ctm,mixes}}{f_{ctm,ref}}}{6}\times 100\%\;[\%]$$

To compare the results of the mechanical performance ratio (*MPR*), Δ*MPR* [–] was used as follows:

(8) $$\Delta MPR=\frac{MPR_{series}}{MPR_{ref}}\;[-]$$

Table 2 presents the results of conducted research.

**Figure 16:**
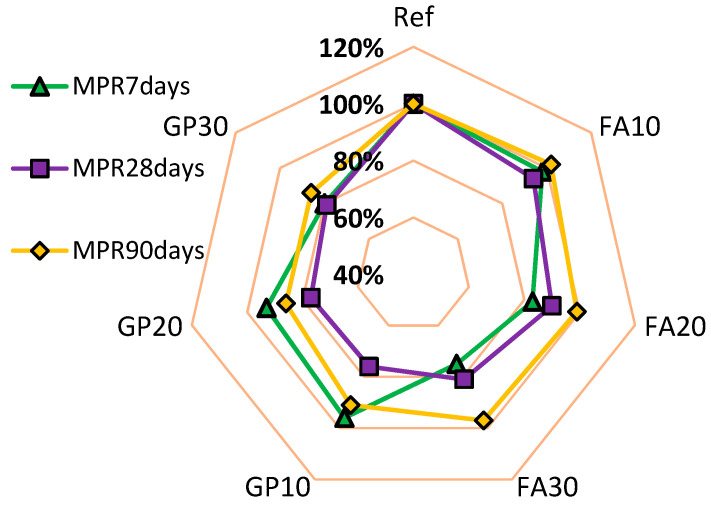
Mechanical performance ratio (*MPR*) value with regards to the type of air-cured cementitious mortar series and the number of days after which the samples were tested.

Figure 16 shows the value of the mechanical performance ratio (*MPR*) with regards to the series of air-cured hardened cementitious mortars and the number of days after which the samples were tested. The *MPR* values for the series with the granite powder are smaller than the *MPR* values for the reference mortar. The *MPR* values for the series with the addition of the fly ash are similar to the *MPR* values for the reference mortar. The mortar with the granite powder has *MPR* values of about 10% lower than the mortar with the fly ash.

### 4.2. Economic Analysis of the Cementitious Composites

The cost of a cementitious mix depends on the cost of its ingredients, which in turn depends on the location and given economic conditions prevailing in the country. In order to determine the costs of materials, which are indicated in Table 3, Polish companies were asked about the costs of individual materials. Table 3 provides the average values. To determine the validity of individual material costs in relation to their importance regarding the composition of the cementitious mix, it was decided to use the effective cost ratio (*ECR*)—the author’s own proposition.

(9) $$c_{ingredient}=uc_{ingredient}\times ingredient\;ratio\;[€]$$

(10) $$c_{mixes}=c_{cement}+c_{GP}+c_{FA}+c_{water}+c_{sand}\;[€]$$

(11) $$ECR=\frac{c_{mixes}}{c_{ref}}\times 100\%\;[\%]$$

(12) $$\Delta ECR=\frac{ECR_{mixes}}{ECR_{ref}}\;[-]$$

**Figure 17:**
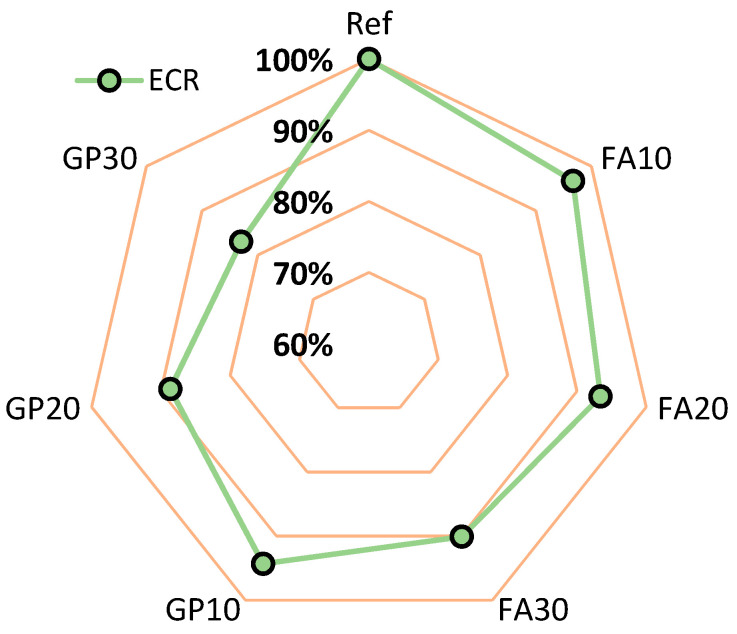
Effective cost ratio (*ECR*) value with regards to the type of cementitious mortar series.

Figure 17 presents the value of the effective cost ratio (*ECR*) with regards to the series of the air-cured hardened cementitious mortars. The ECR value for the series with the granite powder is less than the *ECR* value for the reference mortar. The *ECR* values for the series with the addition of the fly ash are similar to the *ECR* values for the reference mortar. The mortar with the granite powder has *ECR* values of about 5% lower than the mortar with the fly ash.

### 4.3. Combined Economic and Mechanical Performance of the Composites 

Conducting a separate economic and strength analysis does not allow for a proper interpretation of the obtained results, and therefore a combination of these analyzes was performed. Table 4 shows the values of the *MPR* and *ECR* parameters with regards to the series of cementitious mortars.

**Figure 18:**
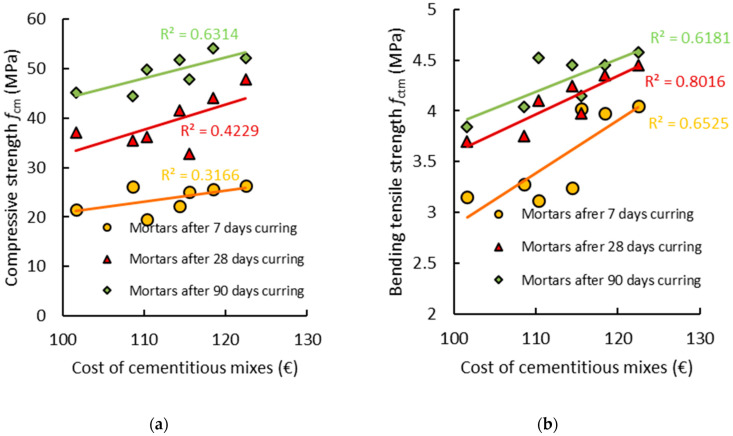
(**a**) Compressive strength (*f_cm_*) and (**b**) bending tensile strength (*f_ctm_*) of the air-cured cementitious mortars in relation to the cost of the cementitious mixes.

Figure 18a,b shows the relationship between the mechanical properties and costs of the cementitious mortars. It can be seen, as expected, that the mechanical properties increase, and therefore a higher price for the cementitious mix should be expected.

**Figure 19:**
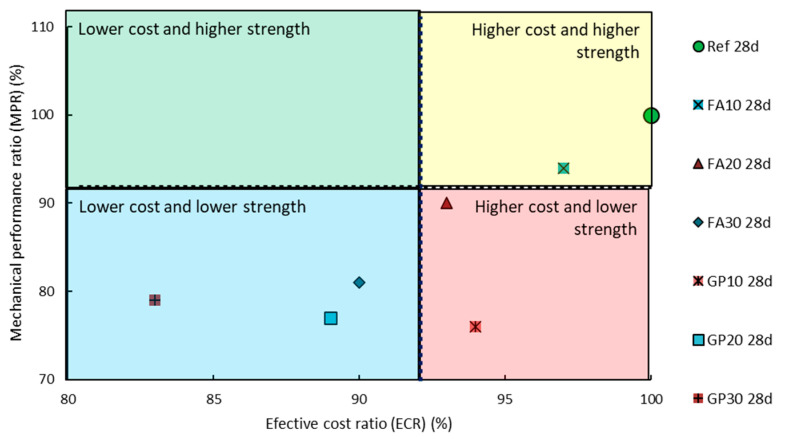
Mechanical performance ratio (*MPR*) and effective cost ratio (*ECR*) values after 28 days with regards to the series of the air-cured hardened cementitious mortars.

**Figure 20:**
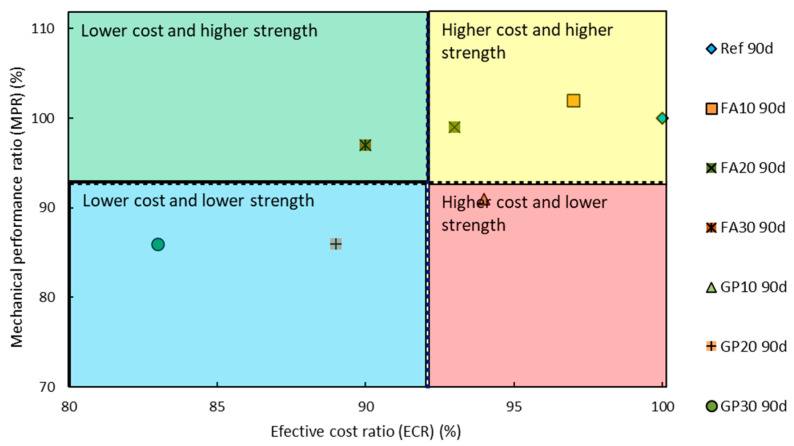
Mechanical performance ratio (*MPR*) and effective cost ratio (*ECR*) values after 90 days with regards to the series of the air-cured hardened cementitious mortars.

Figure 19 and Figure 20 show the division of the cementitious mortars with regards to the mechanical performance ratio (*MPR*) and effective cost ratio (*ECR*) values obtained during the tests. The most optimal choice depends on the situation and should be made by the designer of the building construction. Therefore, the most optimal composition of the mortar was not specified.

## 5. Conclusions

This study allows the comparison of the properties of granite powder waste with the most commonly used supplementary cementitious materials (SCM)—fly ash. It was observed that granite powder has different morphological properties and a different effect on a cement mix when compared to fly ash. However, both additives reduce the mechanical properties of composites, especially in the first 28 days of curing. Nevertheless, it should be stated that the use of granite powder waste as SCM has the potential to improve the sustainability of cementitious mortars. The better cost parameters of mixes with the addition of granite powder, when compared to fly ash, indicate that it is an important additive for cementitious mixes. Moreover, the following observations can be stated:

- Granite powder and fly ash have different morphological properties, whereas the properties of granite powder are similar to the morphology properties of cement;
- The addition of granite powder in cementitious mixes changes the consistency of the mix, becoming denser, whereas fly ash changes the consistency of the mix to be more fluid;
- The addition of granite powder and fly ash causes a decrease in the mechanical properties of cementitious mortars in the first 28 days of curing. After this time, an improvement in the mechanical properties of mortars with the addition of fly ash can be observed. In the case of mortars with the addition of granite powder, this effect was not observed;
- Replacing the cement resulted in a decrease in compressive strength; however, it was observed that with the increased amount of cement replacement with the addition of granite powder (especially in 30% replacement), no significant decrease in compressive strength was detected. This may be indicative of the filler effect of granite powder in cementitious composites;
- It was observed that the increased w/c ratio decreases the compressive strength and bending tensile strength of mortars (compared with reference mortar); it was also observed that the brittleness of mortars decreases with the time of curing, and modification of mortars with the addition of granite powder or fly ash does not change brittleness significantly;
- It was observed that the economic properties of mortars modified with the addition of granite powder are significantly better than those of mortars modified with the addition of fly ash.

It should be stated that granite powder has great potential to be used as supplementary cementitious materials in cementitious composites. However, more research should be conducted to develop the sustainable use of this additive for construction materials.

## Figures and Tables

**Table 1:** Mix design proportions of the mortars.

Series of Mixes [-]	Cement CEM I 42.5R [kg/m^3^]	Water [kg/m^3^]	Fly Ash [kg/m^3^]	Granite Powder [kg/m^3^]	Dried Quartz Sand [kg/m^3^]	Water/Cement Ratio [-]
Ref.	280	140	0	0	840	0.50
FA10	252	140	28	0	840	0.56
FA20	224	140	56	0	840	0.63
FA30	196	140	84	0	840	0.71
GP10	252	140	0	28	840	0.56
GP20	224	140	0	56	840	0.63
GP30	196	140	0	84	840	0.71

**Table 2:** The results of the analysis of the mechanical properties of the cementitious mortars with the addition of the fly ash and granite powder.

Series	*MPR*_7days_	Δ*MPR*_7days_	*MPR*_28days_	Δ*MPR*_28days_	*MPR*_90days_	Δ*MPR*_90days_
Ref	100%	1.00	100%	1.00	100%	1.00
FA10	98%	0.98	94%	0.94	102%	1.02
FA20	83%	0.83	90%	0.90	99%	0.99
FA30	75%	0.75	81%	0.81	97%	0.97
GP10	96%	0.96	76%	0.76	91%	0.91
GP20	93%	0.93	77%	0.77	86%	0.86
GP30	80%	0.80	79%	0.79	86%	0.86

**Table 3:** Economic performance of the properties of the cementitious mortars.

Series	*c_cement_* [€]	*c_water_* [€]	*c_FA_* [€]	*c_GP_* [€]	*c_sand_* [€]	*c_mixes_* [€]	*ECR*	Δ*ECR*
-	-						[%]	[-]
Ref	74.66	2.50	0.00	0.00	45.30	122.46	100	1.00
FA10	67.19	2.50	3.43	0.00	45.30	118.42	97	0.97
FA20	59.73	2.50	6.85	0.00	45.30	114.38	93	0.93
FA30	52.26	2.50	10.28	0.00	45.30	110.34	90	0.90
GP10	67.19	2.50	0.00	0.52	45.30	115.51	94	0.94
GP20	59.73	2.50	0.00	1.03	45.30	108.56	89	0.89
GP30	52.26	2.50	0.00	1.55	45.30	101.61	83	0.83

1. To facilitate the analysis, unit costs were adopted: *uc_cement_* = 74,661 EUR/1000 kg; *uc_water_* = 500 EUR/1000 kg; *uc_FA_* = 3425 EUR/1000 kg; *uc_G_*_P_ = 515 EUR/1000 kg; *uc_sand_* = 1510 EUR/1000 kg, 2. Shipping cost was omitted.

**Table 4:** Mechanical performance ratio (*MPR*) and effective cost ratio (*ECR*) parameters with regards to the cementitious mortar series.

Series	*MPR*_7days_ [%]	*MPR*_28days_ [%]	*MPR*_90days_ [%]	*ECR* [%]
Ref	100	100	100	100
FA10	109	94	102	97
FA20	92	90	99	93
FA30	84	81	97	90
GP10	96	76	91	94
GP20	93	77	86	89
GP30	80	79	86	83

## Data Availability

The data presented in this study are available on request from the corresponding author.
